# Cooperative Computation for Multiuser Task Offloading in Wireless-Powered MEC Systems

**DOI:** 10.3390/s26175568

**Published:** 2026-09-02

**Authors:** Yuan Zheng, Fengxian Tang, Dongqing Li, Yongxue Wang

**Affiliations:** 1 School of Integrated Circuits, Shenzhen Polytechnic University, Shenzhen 518055, China; 2 School of Electronic and Communication Engineering, Shenzhen Polytechnic University, Shenzhen 518055, China

**Keywords:** wireless-powered mobile edge computing, cooperative-computation offloading, helper selection, resource allocation

## Abstract

This paper investigates joint computing and relaying for multiuser task offloading in a wireless-powered mobile edge computing (MEC) system comprising an energy node (EN), an edge server (ES), and multiple energy-harvesting users. One user is selected as the helper for the remaining task users. Each task user partitions its workload among local computing, cooperative computing at the helper, and remote execution at the ES. During a parallel cooperation stage, the helper computes one portion of the uploaded tasks locally while forwarding the remaining portion to the ES and also processes its own task through local computing or edge offloading. The weighted sum computation rate (WSCR) is maximized by jointly optimizing helper selection, task partitioning, time allocation, transmission-energy allocation, and CPU-resource allocation under frame-duration, energy-neutrality, communication, and computation constraints. For each candidate helper, transmission-energy variables are introduced to decouple transmission time and power, and the perspective structure of the achievable-rate functions is exploited to reformulate the continuous resource-allocation problem as an equivalent convex problem. By solving the convex problem for all the candidate helpers, the globally optimal helper selection and resource allocation are obtained. The numerical results show that the proposed joint computing-and-relaying scheme consistently outperforms computing-only, relaying-only, and dedicated-helper cooperation. The performance gain stems from adaptively balancing helper computing and ES processing according to the prevailing communication, computation, and energy bottlenecks.

## 1. Introduction

The rapid growth of wireless sensor networks and Internet of Things (IoT) applications has imposed increasingly stringent requirements on real-time and computation-intensive data processing at energy-constrained wireless devices (WDs). However, many IoT devices, including sensors, wearable terminals, and embedded controllers, are constrained by limited battery capacity and modest on-device computing capability. Mobile edge computing (MEC) alleviates the computing limitation by deploying computing resources at the network edge, enabling WDs to offload intensive tasks to a nearby edge server (ES) for remote execution [1]. Compared with conventional cloud computing, MEC reduces backhaul latency and supports faster responses for delay-sensitive applications. Nevertheless, both local computation and wireless task offloading consume considerable device energy. The finite energy supply of WDs therefore remains a fundamental obstacle to sustainable MEC services in low-power IoT networks.

Wireless-powered MEC integrates radio frequency (RF) wireless power transfer (WPT) with MEC to jointly address the energy and computing limitations of WDs. In this paradigm, an energy transmitter delivers controllable RF energy to the WDs, which use the harvested energy for local task execution or computation offloading. Binary computation offloading was studied in [2], where each task is executed entirely either at the WD or the ES. Partial computation offloading was subsequently considered in [3], allowing each task to be partitioned between local and edge execution. These studies revealed that the computation performance of wireless-powered MEC depends on the coordinated allocation of energy, communication, and computing resources. In particular, extending the WPT duration increases the energy that is available for computation and transmission but leaves less time for task offloading. Meanwhile, the optimal task partition depends jointly on the harvested energy, offloading-channel quality, and local CPU capability. Such coupling makes WPT scheduling, task partitioning, and computation-resource allocation inseparable.

Recent studies have extended wireless-powered MEC toward dynamic resource management and more flexible offloading architectures. Online and learning-based approaches were developed in [4,5,6] to coordinate wireless charging, task execution, and resource allocation under stochastic system dynamics or incomplete future information. Reconfigurable intelligent surfaces were incorporated into wireless-powered MEC in [7] to enhance both energy transfer and task offloading. More flexible multiple-access designs were investigated through successive interference cancellation in [8] and non-orthogonal multiple access in [9]. In addition, data compression was jointly optimized with wireless charging and offloading in [10] to reduce the communication load of raw task data. These studies improve the adaptability and resource efficiency of wireless-powered MEC. However, most of them still rely on direct user-to-ES offloading. When the direct offloading link is weak, a task user may consume excessive transmission time and energy, thereby limiting the achievable computation gain from the harvested energy.

Cooperative-computation offloading provides an effective means of alleviating weak user-to-ES links and limited local computing capability. In [11], computation tasks are partitioned among local computing, helper computing, and ES execution in a multiuser cooperation-assisted wireless-powered MEC system. Opportunistic helper scheduling was investigated in [12], where a nearby user computes part of a task and forwards the remaining part to an MEC server. Helper selection was further coupled with user association, resource-block assignment, task allocation, and computing-resource allocation in [13]. User-centric cooperative offloading was considered in [14], allowing users to obtain communication and computing services from one or multiple MEC access points. Wireless-powered cooperative computing was also integrated with a beyond-diagonal reconfigurable intelligent surface in [15] to enhance energy transfer, task offloading, and cooperative task execution. These studies demonstrate that helper-assisted computing and cooperative offloading can expand the available task-processing resources and mitigate unfavorable direct offloading conditions in multiuser MEC systems.

These multiuser developments build on several foundational models of cooperative-computation offloading. Relay-assisted offloading was investigated in [16] for a two-user wireless-powered MEC system, where one user assists the other by forwarding its offloaded task to an edge cloud without performing cooperative task computation at the assisting user. Joint computation and communication cooperation was considered in [17], where a dedicated helper computes part of the workload of a single task user locally and relays the remaining part to an access point connected to an MEC server. In this dedicated-helper structure, the helper is not selected from the participating users, and multiuser sharing of the helper’s computation and communication resources does not arise. A more closely related wireless-powered model was studied in [18], where a predetermined helping user not only partitions the received workload between local computing and ES forwarding but also processes its own task. Nevertheless, the cooperation relationship is still limited to two users. These studies reveal the complementary roles of computing and relaying cooperation. Helper computing avoids additional helper-to-ES transmission but is constrained by the helper’s CPU capability, whereas relaying exploits the stronger computing capability of the ES at the cost of an additional communication hop and the associated time and energy consumption. Beyond computation offloading and resource allocation, secure and resilient coordination has also received increasing attention in networked intelligent systems. Digital-twin-based resilient consensus control was investigated for UAV systems under attacks in [19], while a data-driven twin-layer approach was developed in [20] for leader-following consensus of nonlinear multi-agent systems under composite attacks.

Despite this progress, joint computing and relaying in a multiuser wireless-powered MEC system has not been fully addressed. The existing wireless-powered cooperative offloading models mainly focus on two-user systems or predetermined cooperation relationships, while the recent helper-assisted MEC studies generally do not consider selecting one energy-harvesting user to simultaneously assist multiple task users and process its own task. In this setting, the helper’s harvested energy, CPU cycles, forwarding time, and transmission energy must be shared among multiple uploaded tasks and its own task. Moreover, helper selection simultaneously changes the EN-to-helper energy-transfer channel, the task-user-to-helper uploading links, the helper-to-ES forwarding link, and the user-specific computation parameters. The helper-computed and ES-forwarded task portions share the same harvested-energy budget, while their corresponding computing and forwarding operations proceed in parallel. Consequently, helper selection, task partitioning, and continuous resource allocation are tightly coupled and cannot be optimized independently.

**Figure 1:**
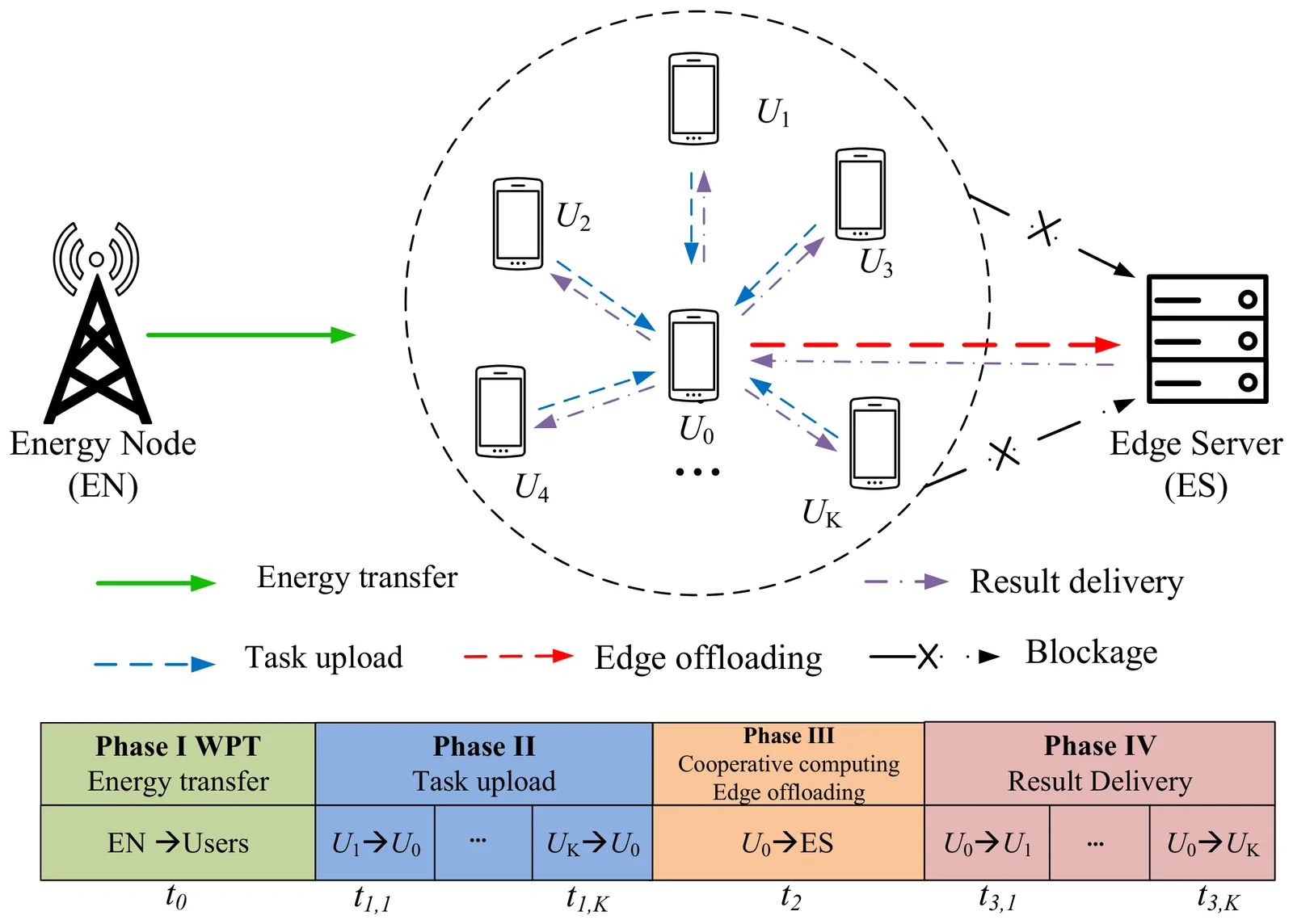
System model and transmission protocol of the proposed system.

Motivated by these observations, this paper investigates a helper-assisted multiuser wireless-powered MEC system consisting of one EN, one ES, and multiple energy-harvesting users. As illustrated in Figure 1, one user is selected as the helper, while the remaining users act as task users. Each task user computes part of its task locally and uploads the remaining part to the helper. During a parallel cooperation stage, the helper computes one portion of the uploaded tasks locally and forwards the other portion to the ES for remote execution while also processing its own task through local computing or edge offloading. The helper subsequently delivers the corresponding computation results to the task users. Accordingly, each task user’s workload can be adaptively partitioned among local computing, cooperative computing at the helper, and remote processing at the ES according to the prevailing communication, computation, and energy conditions.

The main contributions of this paper are summarized as follows.

- This paper develops a multiuser helper-selection architecture that jointly exploits computing and relaying cooperation in a wireless-powered MEC system. Unlike two-user or dedicated-helper cooperation models, one energy-harvesting user is dynamically selected as the helper for the remaining task users while also processing its own task. During a parallel cooperation stage, the selected helper computes part of the uploaded tasks locally and forwards the remaining part to the ES. Consequently, its harvested energy, CPU resources, forwarding time, and transmission energy are shared among multiple uploaded tasks and its own task. This architecture enables flexible task partitioning between helper computing and ES processing under coupled multiuser resource constraints.
- A weighted sum computation rate (WSCR) maximization problem is formulated by jointly optimizing helper selection, task partitioning, time allocation, transmission-energy allocation, and CPU-resource allocation under frame-duration, energy-neutrality, communication, and computation constraints. For each candidate helper, transmission-energy variables are introduced to decouple transmission time and power, while the perspective structures of the communication-rate and computation-energy functions are exploited to transform the continuous resource-allocation problem into an equivalent convex problem. By solving this problem for all the candidate helpers, the globally optimal helper selection and resource allocation are obtained.
- The numerical results reveal the complementary roles of computing and relaying cooperation under different energy, communication, and computation conditions. The proposed joint computing-and-relaying scheme consistently outperforms computing-only, relaying-only, and dedicated-helper cooperation, while the relative performance of the benchmark schemes varies with the prevailing system bottleneck. The performance gain results from adaptively allocating the uploaded tasks between helper computing and ES processing and selecting the helper according to the network conditions.

The remainder of this paper is organized as follows. Section 2 presents the system model and the proposed helper-assisted offloading protocol. Section 3 characterizes the computation performance and formulates the WSCR maximization problem. Section 4 develops the optimal helper-selection and resource-allocation method. Section 5 presents the simulation results, and Section 6 concludes the paper.

## 2. System Model

### 2.1. Network Model

As shown in Figure 1, this paper considers a multiuser wireless-powered MEC system consisting of one EN, one ES, and *N* energy-harvesting users indexed by N≜{1,…,N}. One user $U_{n}$, n∈N is selected as the helper to provide computing and relaying assistance for the remaining users. For each candidate helper, the system reindexes $U_{n}$ as $U_{0}$ and denotes the remaining K=N−1 task users by K≜{1,…,K}. The paper further defines $K_{0}≜\left\{0\right\}\cup K$. All user-specific channel, energy-harvesting, computation, and priority parameters are relabeled accordingly. All nodes are equipped with a single antenna.

The EN continuously draws energy from a stable power supply and transfers radio frequency energy to the users. The users utilize the harvested energy for task computation and wireless transmission, whereas the ES has sufficient energy and computing resources to execute the offloaded tasks. Each user is equipped with a sufficiently large rechargeable energy buffer. The system is assumed to operate in a steady-state regime in which a certain amount of energy harvested in previous frames is available at the beginning of each frame. This stored energy supports transient energy expenditure before the current-frame WPT energy becomes fully available, thereby avoiding explicit intra-frame energy-causality constraints. Nevertheless, frame-level energy neutrality is strictly imposed such that the total energy consumed by each user in one frame cannot exceed the energy harvested during that frame. Therefore, the stored energy is not continuously depleted across frames. This model is appropriate for buffer-equipped wireless-powered devices operating over consecutive frames; an initially empty energy buffer would require additional cumulative intra-frame energy-causality constraints. This system considers helper-assisted computation offloading, where the task users do not directly offload tasks to the ES. Instead, $U_{0}$ receives the offloaded tasks, computes one portion of each uploaded task locally, and forwards the remaining portion to the ES. Accordingly, the task of each $U_{k}$, k∈K can be executed locally at $U_{k}$, cooperatively at $U_{0}$, or remotely at the ES. Meanwhile, $U_{0}$ also processes its own task through local computation or edge offloading.

Let ${\tilde{g}}_{i}$, $i\in K_{0}$ denote the complex channel coefficient from the EN to $U_{i}$. Let ${\tilde{h}}_{k}$ denote the complex channel coefficient between $U_{k}$ and $U_{0}$, and let ${\tilde{h}}_{0}$ denote that between $U_{0}$ and the ES. The corresponding channel power gains are given by

(1) $$g_{i}={\left|{\tilde{g}}_{i}\right|}^{2},\;i\in K_{0},$$

(2) $$h_{k}={\left|{\tilde{h}}_{k}\right|}^{2},\;k\in K,$$

and

(3) $$h_{0}={\left|{\tilde{h}}_{0}\right|}^{2}.$$

The user-to-user channels are assumed to be reciprocal. All wireless links follow a distance-dependent path-loss model and are quasi-static within each transmission frame, while the channel conditions may vary across frames. The instantaneous channel gains are assumed to be perfectly available at the system controller at the beginning of each frame such that helper selection and resource allocation can be updated on a frame-by-frame basis. Channel-estimation errors and the associated signaling overhead are not modeled in the present framework; accounting for these effects would require robust resource allocation and explicit modeling of channel-acquisition overhead. For high-mobility scenarios with significant channel variations within one frame, the quasi-static assumption may no longer be accurate, and mobility-aware prediction or robust helper selection would be required.

The ES is assumed to have sufficiently high computing capability and transmit power, together with a high-capacity downlink to $U_{0}$. Therefore, the task-execution time at the ES and the result-return time from the ES to $U_{0}$ are neglected. In contrast, the result delivery from $U_{0}$ to the task users is explicitly modeled because $U_{0}$ is powered by harvested energy. A fixed circuit energy consumption $E_{i}^{\mathrm{cir}}$ is considered for each user $U_{i}$ to account for signal reception, control signaling, and circuit operation.

### 2.2. Transmission Protocol

The system considers a transmission frame of duration *T*, whose time allocation is illustrated in Figure 1. At the beginning of each frame, the EN transfers wireless energy to all users with a fixed transmit power $P_{0}$ for a duration of $t_{0}$.

Following the WPT period, the task users sequentially upload their offloaded task portions to $U_{0}$ using time-division multiple access. During this phase, $U_{0}$ operates only as an information receiver, and energy harvesting from the task users’ uplink transmissions is not considered. Let $t_{1,k}$ denote the task-upload time allocated to $U_{k}$, k∈K. The total task-upload duration is

(4) $$t_{1}=\sum_{k\in K}t_{1,k}.$$

After receiving the uploaded tasks, $U_{0}$ enters a parallel cooperation stage during which it computes the task portions assigned to the helper while forwarding the remaining portions to the ES. Let $t_{2,k}^{c}$ denote the time used by $U_{0}$ to compute the task portion of $U_{k}$. Since the processor of $U_{0}$ can execute only one task at a time, the total cooperative-computation time is

(5) $$t_{2}^{c}=\sum_{k\in K}t_{2,k}^{c}.$$

Let $t_{2,k}^{f}$ denote the time used by $U_{0}$ to forward the task portion of $U_{k}$ to the ES, and let $t_{2,0}^{f}$ denote the time used by $U_{0}$ to offload its own task. The total helper-to-ES transmission time is

(6) $$t_{2}^{f}=t_{2,0}^{f}+\sum_{k\in K}t_{2,k}^{f}.$$

The processor and wireless transceiver of $U_{0}$ are modeled as separate functional units and are allowed to operate concurrently. Therefore, the single-processor constraint prevents $U_{0}$ from executing multiple computation tasks simultaneously but does not prevent CPU computation from proceeding in parallel with wireless transmission. Hence, cooperative computing and edge offloading can overlap in time, and the duration of this stage is determined by the longer of the two operations:

(7) $$t_{2}=\max\left\{t_{2}^{c},t_{2}^{f}\right\}.$$

After helper computing and ES processing are completed, the ES returns its computation results to $U_{0}$. The helper aggregates the locally generated results and those returned by the ES and then sequentially delivers the corresponding results to the task users. Let $t_{3,k}$ denote the result-delivery time from $U_{0}$ to $U_{k}$. The total result-delivery duration is

(8) $$t_{3}=\sum_{k\in K}t_{3,k}.$$

Accordingly, the overall time allocation satisfies

(9) $$t_{0}+t_{1}+t_{2}+t_{3}\leq T,$$

where all time-allocation variables are nonnegative.

## 3. Computation Model and Problem Formulation

During the WPT period, the EN broadcasts wireless energy with a fixed transmit power $P_{0}$ for a duration of $t_{0}$. Let $\eta_{i}\in \left(0,1\right]$ denote the energy-conversion efficiency of $U_{i}$. The energy harvested by $U_{i}$ is

(10) $$Q_{i}=\eta_{i}P_{0}g_{i}t_{0},\;i\in K_{0}.$$

The task users use their harvested energy for local computation and task uploading, whereas the helper $U_{0}$ uses its harvested energy for local computation, cooperative computing, edge offloading, and result delivery. It is assumed that each user has a sufficiently large task-input buffer such that the number of processed task bits is determined by the available communication, computation, and energy resources.

Under partial computation offloading, the task of each $U_{k}$, k∈K is partitioned into three portions. Let $b_{k,k}$, $b_{k,0}$, and $b_{k,E}$ denote the numbers of task bits computed locally at $U_{k}$, cooperatively at $U_{0}$, and remotely at the ES, respectively. The total number of processed task bits of $U_{k}$ is

(11) $$b_{k}=b_{k,k}+b_{k,0}+b_{k,E},\;k\in K.$$

The task of $U_{0}$ is partitioned into a locally computed portion and an edge-computed portion. Let $b_{0,0}$ and $b_{0,E}$ denote the corresponding numbers of task bits. The total number of processed task bits of $U_{0}$ is

(12) $$b_{0}=b_{0,0}+b_{0,E}.$$

### 3.1. Local Computation

Let $\phi_{i}$ denote the number of CPU cycles required to process one input bit of the task generated by $U_{i}$, and let $\kappa_{i}$ denote the effective switched-capacitance coefficient of the processor at $U_{i}$.

The processor and wireless transceiver of each task user are assumed to operate simultaneously. Therefore, each $U_{k}$ can perform local computation throughout the entire frame while participating in wireless transmission. Let $f_{k}\in \left[0,f_{k}^{\max}\right]$ denote the CPU frequency of $U_{k}$. The number of task bits locally computed by $U_{k}$ is

(13) $$b_{k,k}=\frac{f_{k}T}{\phi_{k}},\;k\in K,$$

and the corresponding energy consumption is

(14) $$E_{k,k}^{\mathrm{loc}}=\kappa_{k}f_{k}^{3}T,\;k\in K.$$

The helper $U_{0}$ allocates part of its computing time to processing its own task. Let $t_{0}^{\mathrm{loc}}$ and $f_{0}^{\mathrm{loc}}$ denote the corresponding computation time and CPU frequency, respectively, where

$$0\leq f_{0}^{\mathrm{loc}}\leq f_{0}^{\max}.$$

The number of task bits locally computed by $U_{0}$ is

(15) $$b_{0,0}=\frac{f_{0}^{\mathrm{loc}}t_{0}^{\mathrm{loc}}}{\phi_{0}},$$

and the corresponding energy consumption is

(16) $$E_{0,0}^{\mathrm{loc}}=\kappa_{0}{\left(f_{0}^{\mathrm{loc}}\right)}^{3}t_{0}^{\mathrm{loc}}.$$

### 3.2. Cooperative Computing and Relaying

During $t_{1,k}$, task user $U_{k}$ uploads the task portions assigned to $U_{0}$ and the ES to the helper. Let $p_{1,k}$ denote the corresponding transmit power. Since both portions must first be received by $U_{0}$, the task-upload constraint is

(17) $$b_{k,0}+b_{k,E}\leq t_{1,k}B\log_{2}\left(1+\frac{h_{k}p_{1,k}}{\Gamma N_{0}}\right),\;k\in K,$$

where *B* denotes the system bandwidth, $N_{0}$ denotes the receiver noise power, and Γ≥1 denotes the SNR gap from the Shannon capacity caused by practical modulation and coding.

The corresponding transmission-energy consumption of $U_{k}$ is

(18) $$E_{k}^{\mathrm{off}}=t_{1,k}p_{1,k},\;k\in K.$$

After receiving the uploaded task data, $U_{0}$ forwards the $b_{k,E}$ bits assigned to the ES. Let $p_{2,k}^{f}$ denote the forwarding power during $t_{2,k}^{f}$. The corresponding helper-to-ES transmission constraint is

(19) $$b_{k,E}\leq t_{2,k}^{f}B\log_{2}\left(1+\frac{h_{0}p_{2,k}^{f}}{\Gamma N_{0}}\right),\;k\in K.$$

In addition, $U_{0}$ offloads $b_{0,E}$ bits of its own task to the ES during $t_{2,0}^{f}$. Let $p_{2,0}^{f}$ denote the corresponding transmit power. Thus,

(20) $$b_{0,E}\leq t_{2,0}^{f}B\log_{2}\left(1+\frac{h_{0}p_{2,0}^{f}}{\Gamma N_{0}}\right).$$

Accordingly, the total energy consumed by $U_{0}$ for forwarding the task users’ data and offloading its own task is

(21) $$E_{0}^{\mathrm{off}}=t_{2,0}^{f}p_{2,0}^{f}+\sum_{k\in K}t_{2,k}^{f}p_{2,k}^{f}.$$

Besides forwarding the ES-assigned task portions, $U_{0}$ computes $b_{k,0}$ bits for each task user $U_{k}$. Let $f_{k,0}$ denote the CPU frequency used by $U_{0}$ to process the task of $U_{k}$ during $t_{2,k}^{c}$, where

$$0\leq f_{k,0}\leq f_{0}^{\max}.$$

The number of task bits cooperatively computed at $U_{0}$ satisfies

(22) $$b_{k,0}=\frac{f_{k,0}t_{2,k}^{c}}{\phi_{k}},\;k\in K.$$

The corresponding computation-energy consumption is

(23) $$E_{k,0}^{c}=\kappa_{0}f_{k,0}^{3}t_{2,k}^{c},\;k\in K.$$

Since the processor of $U_{0}$ can execute only one computation task at a time, the cooperative-computation tasks are processed sequentially. The local computation of $U_{0}$’s own task is not restricted to the cooperation stage and can be scheduled during CPU-idle intervals over the frame. Therefore, $t_{0}^{\mathrm{loc}}$ denotes the aggregate CPU time used for $U_{0}$’s own task, and the nonoverlap of its own local computation and the cooperative-computation tasks is enforced by

(24) $$t_{0}^{\mathrm{loc}}+\sum_{k\in K}t_{2,k}^{c}\leq T.$$

After helper computing and ES processing are completed, $U_{0}$ delivers the corresponding computation results to the task users. Let $\nu_{k}\in \left(0,1\right]$ denote the output-to-input data ratio of the nonlocally processed task of $U_{k}$. The same ratio is assumed for the task portions processed at the helper and the ES. Let $p_{3,k}$ denote the transmit power used by $U_{0}$ during $t_{3,k}$. The result-delivery constraint is

(25) $$\nu_{k}\left(b_{k,0}+b_{k,E}\right)\leq t_{3,k}B\log_{2}\left(1+\frac{h_{k}p_{3,k}}{\Gamma N_{0}}\right),\;k\in K.$$

The total energy consumed by $U_{0}$ for result delivery is

(26) $$E_{0}^{d}=\sum_{k\in K}t_{3,k}p_{3,k}.$$

Here, $E_{i}^{\mathrm{cir}}$ denotes the fixed circuit energy consumption of $U_{i}$ in each transmission frame. Accordingly, the energy-neutrality constraint of each task user is

(27) $$E_{k,k}^{\mathrm{loc}}+E_{k}^{\mathrm{off}}+E_{k}^{\mathrm{cir}}\leq Q_{k},\;k\in K.$$

whereas the energy-neutrality constraint of $U_{0}$ is

(28) $$E_{0,0}^{\mathrm{loc}}+\sum_{k\in K}E_{k,0}^{c}+E_{0}^{\mathrm{off}}+E_{0}^{d}+E_{0}^{\mathrm{cir}}\leq Q_{0}.$$

### 3.3. Problem Formulation

The computation rates of task user $U_{k}$ and helper user $U_{0}$ are respectively defined as

(29) $$R_{k}=\frac{b_{k,k}+b_{k,0}+b_{k,E}}{T},\;k\in K,$$

and

(30) $$R_{0}=\frac{b_{0,0}+b_{0,E}}{T}.$$

For a given helper candidate $U_{n}$, which is reindexed as $U_{0}$, task partitioning, time allocation, transmission powers, and CPU frequencies are jointly optimized to maximize the weighted sum computation rate. Define

(31) $$b≜\left\{b_{0,0},b_{0,E}\right\}\cup {\left\{b_{k,k},b_{k,0},b_{k,E}\right\}}_{k\in K},$$

(32) $$t≜\left\{t_{0},t_{2},t_{0}^{\mathrm{loc}},t_{2,0}^{f}\right\}\cup {\left\{t_{1,k},t_{2,k}^{c},t_{2,k}^{f},t_{3,k}\right\}}_{k\in K},$$

(33) $$p≜\left\{p_{2,0}^{f}\right\}\cup {\left\{p_{1,k},p_{2,k}^{f},p_{3,k}\right\}}_{k\in K},$$

and

(34) $$f≜\left\{f_{0}^{\mathrm{loc}}\right\}\cup {\left\{f_{k},f_{k,0}\right\}}_{k\in K}.$$

The resource-allocation problem for candidate helper $U_{n}$ is formulated as

(35) $$\begin{array}{cc} \left(P1\right):\;\underset{b,t,p,f}{\mathrm{maximize}}\; & \sum_{i\in K_{0}}w_{i}R_{i}\\ \mathrm{subject}\;\mathrm{to}\; & t_{0}+\sum_{k\in K}t_{1,k}+t_{2}+\sum_{k\in K}t_{3,k}\leq T,\\ \; & \sum_{k\in K}t_{2,k}^{c}\leq t_{2},\;t_{2,0}^{f}+\sum_{k\in K}t_{2,k}^{f}\leq t_{2},\\ \; & (13),\;(15),\;(17),\;(19),\;(20),\;(22),\;(24),\;(25),\;(27)\;\mathrm{and}\;(28),\\ \; & b_{k,k},b_{k,0},b_{k,E}\geq 0,\;k\in K,\\ \; & b_{0,0},b_{0,E}\geq 0,\;t⪰0,\;p⪰0,\\ \; & 0\leq f_{k}\leq f_{k}^{\max},\;k\in K,\\ \; & 0\leq f_{0}^{\mathrm{loc}}\leq f_{0}^{\max},\;0\leq f_{k,0}\leq f_{0}^{\max},\;k\in K. \end{array}$$

The two constraints involving $t_{2}$ constitute the epigraph representation of the parallel cooperation duration defined in (7). Specifically, they ensure that $t_{2}$ is no smaller than either the total cooperative-computation time or the total helper-to-ES transmission time.

Here, $w_{i}>0$ denotes the computation-priority weight associated with the original identity of user $U_{i}$. When $U_{n}$ is selected as the candidate helper, the weights are relabeled consistently with the corresponding users and normalized such that

(36) $$\sum_{i\in K_{0}}w_{i}=1.$$

Although (P1) maximizes the WSCR rather than an energy-efficiency metric, the energy expenditure of each user is strictly constrained by the harvested-energy budgets in (27) and (28). Hence, the computation-rate improvement is achieved through efficient allocation of the available harvested energy rather than unrestricted energy consumption. For IoT applications where energy efficiency is the primary concern, the framework can be extended by adopting an energy-efficiency objective or an objective that jointly accounts for computation performance and energy consumption.

Let $V_{n}^{★}$ denote the optimal objective value of problem (P1). Problem (P1) is nonconvex because of the coupled time–power terms in the communication-rate constraints and the coupled computation-time–CPU-frequency terms in the computation model. An equivalent convex reformulation is developed in the next section.

## 4. Optimal Solution

### 4.1. Resource Allocation for a Given Helper

For a given helper candidate $U_{n}$, problem (P1) is first transformed into an equivalent convex optimization problem. To eliminate the coupling between transmission time and power, define the transmission-energy variables

(37) $$\begin{array}{cc} e_{1,k} & =t_{1,k}p_{1,k},\;k\in K, \end{array}$$

(38) $$\begin{array}{cc} e_{2,k}^{f} & =t_{2,k}^{f}p_{2,k}^{f},\;k\in K, \end{array}$$

(39) $$\begin{array}{cc} e_{2,0}^{f} & =t_{2,0}^{f}p_{2,0}^{f}, \end{array}$$

(40) $$\begin{array}{cc} e_{3,k} & =t_{3,k}p_{3,k},\;k\in K. \end{array}$$

For notational convenience, define

(41) $$e≜\left\{e_{2,0}^{f}\right\}\cup {\left\{e_{1,k},e_{2,k}^{f},e_{3,k}\right\}}_{k\in K}.$$

The transmission-rate function is further defined as

(42) $$R\left(t,e;h\right)≜tB\log_{2}\left(1+\frac{he}{\Gamma N_{0}t}\right).$$

Its closed extension at t=0 is defined as

(43) R(0,e;h)=0,e≥0.

When the transmission time is zero, any positive energy allocation produces no task transmission. Hence, an optimal solution can always be chosen with e=0 whenever the corresponding transmission time is zero.

Using the transmission-energy variables, the task-upload, forwarding, edge-offloading, and result-delivery constraints can be respectively rewritten as

(44) $$b_{k,0}+b_{k,E}\leq R\left(t_{1,k},e_{1,k};h_{k}\right),\;k\in K,$$

(45) $$b_{k,E}\leq R\left(t_{2,k}^{f},e_{2,k}^{f};h_{0}\right),\;k\in K,$$

(46) $$b_{0,E}\leq R\left(t_{2,0}^{f},e_{2,0}^{f};h_{0}\right),$$

and

(47) $$\nu_{k}\left(b_{k,0}+b_{k,E}\right)\leq R\left(t_{3,k},e_{3,k};h_{k}\right),\;k\in K.$$

Next, the CPU-frequency variables are eliminated using (13), (15) and (22). The local-computation energy consumption of task user $U_{k}$ can be expressed as

(48) $$E_{k,k}^{\mathrm{loc}}=\frac{\kappa_{k}\phi_{k}^{3}b_{k,k}^{3}}{T^{2}},\;k\in K.$$

The energy consumed by $U_{0}$ for computing its own task becomes

(49) $$E_{0,0}^{\mathrm{loc}}=\frac{\kappa_{0}\phi_{0}^{3}b_{0,0}^{3}}{{\left(t_{0}^{\mathrm{loc}}\right)}^{2}},$$

whereas the energy consumed by $U_{0}$ for cooperatively computing the task of $U_{k}$ becomes

(50) $$E_{k,0}^{c}=\frac{\kappa_{0}\phi_{k}^{3}b_{k,0}^{3}}{{\left(t_{2,k}^{c}\right)}^{2}},\;k\in K.$$

The above computation-energy functions are interpreted in the extended-value sense. Each function equals zero when both the task size and the corresponding computation time are zero and equals positive infinity when the computation time is zero while the task size is positive.

The CPU-frequency constraints are equivalently rewritten as

(51) $$\phi_{k}b_{k,k}\leq f_{k}^{\max}T,\;k\in K,$$

(52) $$\phi_{0}b_{0,0}\leq f_{0}^{\max}t_{0}^{\mathrm{loc}},$$

and

(53) $$\phi_{k}b_{k,0}\leq f_{0}^{\max}t_{2,k}^{c},\;k\in K.$$

Accordingly, the energy-neutrality constraint of each task user becomes

(54) $$\frac{\kappa_{k}\phi_{k}^{3}b_{k,k}^{3}}{T^{2}}+e_{1,k}+E_{k}^{\mathrm{cir}}\leq \eta_{k}P_{0}g_{k}t_{0},\;k\in K,$$

whereas the energy-neutrality constraint of the helper becomes

(55) $$\frac{\kappa_{0}\phi_{0}^{3}b_{0,0}^{3}}{{\left(t_{0}^{\mathrm{loc}}\right)}^{2}}+\sum_{k\in K}\frac{\kappa_{0}\phi_{k}^{3}b_{k,0}^{3}}{{\left(t_{2,k}^{c}\right)}^{2}}+e_{2,0}^{f}+\sum_{k\in K}e_{2,k}^{f}+\sum_{k\in K}e_{3,k}+E_{0}^{\mathrm{cir}}\leq \eta_{0}P_{0}g_{0}t_{0}.$$

Using the above transformations, problem (P1) is equivalently reformulated as

(56) $$\begin{array}{cc} \left(P2\right):\;\underset{b,t,e}{\mathrm{maximize}}\; & \frac{1}{T}\left[w_{0}\left(b_{0,0}+b_{0,E}\right)+\sum_{k\in K}w_{k}\left(b_{k,k}+b_{k,0}+b_{k,E}\right)\right]\\ \mathrm{subject}\;\mathrm{to}\; & b_{k,0}+b_{k,E}\leq R\left(t_{1,k},e_{1,k};h_{k}\right),\;k\in K,\\ \; & b_{k,E}\leq R\left(t_{2,k}^{f},e_{2,k}^{f};h_{0}\right),\;k\in K,\\ \; & b_{0,E}\leq R\left(t_{2,0}^{f},e_{2,0}^{f};h_{0}\right),\\ \; & \nu_{k}\left(b_{k,0}+b_{k,E}\right)\leq R\left(t_{3,k},e_{3,k};h_{k}\right),\;k\in K,\\ \; & \frac{\kappa_{k}\phi_{k}^{3}b_{k,k}^{3}}{T^{2}}+e_{1,k}+E_{k}^{\mathrm{cir}}\leq \eta_{k}P_{0}g_{k}t_{0},\;k\in K,\\ \; & \frac{\kappa_{0}\phi_{0}^{3}b_{0,0}^{3}}{{\left(t_{0}^{\mathrm{loc}}\right)}^{2}}+\sum_{k\in K}\frac{\kappa_{0}\phi_{k}^{3}b_{k,0}^{3}}{{\left(t_{2,k}^{c}\right)}^{2}}+e_{2,0}^{f}+\sum_{k\in K}e_{2,k}^{f}+\sum_{k\in K}e_{3,k}+E_{0}^{\mathrm{cir}}\leq \eta_{0}P_{0}g_{0}t_{0},\\ \; & t_{0}+\sum_{k\in K}t_{1,k}+t_{2}+\sum_{k\in K}t_{3,k}\leq T,\\ \; & \sum_{k\in K}t_{2,k}^{c}\leq t_{2},\;t_{2,0}^{f}+\sum_{k\in K}t_{2,k}^{f}\leq t_{2},\;t_{0}^{\mathrm{loc}}+\sum_{k\in K}t_{2,k}^{c}\leq T,\\ \; & \phi_{k}b_{k,k}\leq f_{k}^{\max}T,\phi_{0}b_{0,0}\leq f_{0}^{\max}t_{0}^{\mathrm{loc}},\phi_{k}b_{k,0}\leq f_{0}^{\max}t_{2,k}^{c},\;k\in K,\\ \; & b_{k,k},b_{k,0},b_{k,E}\geq 0,\;k\in K,b_{0,0},b_{0,E}\geq 0,\\ \; & t⪰0,\;e⪰0. \end{array}$$

**Proposition** **1.** *For any given helper candidate $U_{n}$, problem (P2) is a convex optimization problem.*

**Proof.**  The objective function of (P2) is affine. The function R(t,e;h) is the perspective of the concave function

(57) $$B\log_{2}\left(1+\frac{hx}{\Gamma N_{0}}\right)$$

and is therefore jointly concave in (t,e). Consequently, the feasible sets defined by the communication constraints in (56) are convex.

Moreover, let $f\left(x\right)=x^{3}$ for x≥0. Since $f^{\prime \prime }\left(x\right)=6x\geq 0$, f(x) is convex on its domain. For b≥0 and t>0,

(58) $$\frac{b^{3}}{t^{2}}=t{\left(\frac{b}{t}\right)}^{3}=tf\left(\frac{b}{t}\right),$$

which is the perspective of the convex function $f\left(x\right)=x^{3}$ and is therefore jointly convex in (b,t). Together with the extended-value definition given above, i.e., zero at (b,t)=(0,0) and positive infinity for b>0 and t=0, this function forms a closed convex perspective over its entire domain. Hence, the computation-energy terms in the energy-neutrality constraints of (P2) are convex. All the remaining constraints are affine. Therefore, problem (P2) is convex, which completes the proof.    □

For a given helper candidate $U_{n}$, problem (P2) can be solved globally using an interior-point method. Let

(59) $$\left(b_{n}^{★},t_{n}^{★},e_{n}^{★}\right)$$

denote its optimal solution. For notational simplicity, the candidate index *n* is omitted from the following recovery expressions.

The optimal transmission powers are recovered as

(60) $$p_{1,k}^{★}=\frac{e_{1,k}^{★}}{t_{1,k}^{★}},\;k\in K,$$

(61) $$p_{2,k}^{f★}=\frac{e_{2,k}^{f★}}{t_{2,k}^{f★}},\;k\in K,$$

(62) $$p_{2,0}^{f★}=\frac{e_{2,0}^{f★}}{t_{2,0}^{f★}},$$

and

(63) $$p_{3,k}^{★}=\frac{e_{3,k}^{★}}{t_{3,k}^{★}},\;k\in K.$$

Similarly, the optimal CPU frequencies are recovered as

(64) $$f_{k}^{★}=\frac{\phi_{k}b_{k,k}^{★}}{T},\;k\in K,$$

(65) $$f_{0}^{\mathrm{loc}★}=\frac{\phi_{0}b_{0,0}^{★}}{t_{0}^{\mathrm{loc}★}},$$

and

(66) $$f_{k,0}^{★}=\frac{\phi_{k}b_{k,0}^{★}}{t_{2,k}^{c★}},\;k\in K.$$

If a transmission-time variable equals zero, the corresponding transmitted task size is zero, and the associated transmission energy and transmit power are set to zero without loss of optimality. Similarly, if a computation-time variable equals zero, the corresponding computed task size and CPU frequency are defined as zero.

### 4.2. Helper Selection

Let $V_{n}^{★}$ denote the optimal WSCR obtained by solving problem (P2) when $U_{n}$ is selected as the helper. The optimal helper is determined by

(67) $$n^{★}=\arg\max_{n\in N}V_{n}^{★}.$$

Accordingly, the globally optimal resource allocation is given by

(68) $$\left(b^{★},t^{★},e^{★}\right)=\left(b_{n^{★}}^{★},t_{n^{★}}^{★},e_{n^{★}}^{★}\right),$$

and the corresponding transmit powers and CPU frequencies are recovered according to (60)–(66).

The joint helper-selection and resource-allocation procedure is summarized in Algorithm 1.

**Proposition** **2.** *Algorithm 1 obtains a globally optimal solution to the joint helper-selection and resource-allocation problem.*

**Proof.**  For any fixed helper candidate $U_{n}$, the transformed problem (P2) is equivalent to the corresponding continuous resource-allocation problem, with $U_{n}$ selected as the helper. By Proposition 1, problem (P2) is convex and can be solved globally. Let $V_{n}^{★}$ denote its optimal WSCR. Any feasible solution of the original joint problem must select exactly one helper $U_{n}$, and its objective value cannot exceed $V_{n}^{★}$ for that candidate. Therefore, letting $V^{★}$ denote the optimal value of the original joint problem, $V^{★}\leq \max_{n\in N}V_{n}^{★}.$ Conversely, Algorithm 1 evaluates every feasible helper candidate and selects $n^{★}$ according to (67). The globally optimal resource allocation associated with $U_{n^{★}}$ is feasible for the original joint problem and achieves $\max_{n\in N}V_{n}^{★}$. Hence, $V^{★}=\max_{n\in N}V_{n}^{★}.$ Therefore, the resulting helper selection and resource allocation are globally optimal, which completes the proof.    □

**Algorithm 1** Optimal Helper Selection and Resource Allocation
   **Input:** Channel gains, energy-harvesting parameters, computation parameters, priority weights, and the user set N.  **Output:** Optimal helper index $n^{★}$, task-partitioning vector $b^{★}$, time allocation $t^{★}$, transmit-power allocation $p^{★}$, CPU-frequency allocation $f^{★}$, and maximum WSCR $V_{\mathrm{best}}$.
  1:Set $V_{\mathrm{best}}=-\infty$ and $n^{★}=0$.  2:**for** each n∈N **do**  3:      Select $U_{n}$ as the candidate helper and reindex it as $U_{0}$.  4:      Relabel the corresponding channel, energy-harvesting, computation, and priority parameters.  5:      Construct and solve (P2) using an interior-point method, and obtain $b_{n}^{★}$, $t_{n}^{★}$, $e_{n}^{★}$, and $V_{n}^{★}$.  6:      **if** $V_{n}^{★}>V_{\mathrm{best}}$ **then**  7:             Set $V_{\mathrm{best}}=V_{n}^{★}$ and $n^{★}=n$.  8:             Set $b^{★}=b_{n}^{★}$, $t^{★}=t_{n}^{★}$, and $e^{★}=e_{n}^{★}$.  9:      **end if**10:
**end for**
11:Recover $p^{★}$ according to (60)–(63).12:Recover $f^{★}$ according to (64)–(66).13:**return** $n^{★}$, $b^{★}$, $t^{★}$, $p^{★}$, $f^{★}$, and $V_{\mathrm{best}}$.


For each candidate helper, problem (P2) contains 10K+7 scalar optimization variables, and its number of constraints grows linearly with K=N−1. Since Algorithm 1 solves one convex subproblem for each of the *N* candidate helpers, its computational burden increases with both the number and size of the subproblems. Algorithm 1 is designed to obtain the globally optimal solution and therefore primarily serves as an exact optimal-performance benchmark rather than a real-time optimization method. For practical online implementation, the candidate-helper set can be reduced using slowly varying information, such as large-scale channel gains, user locations, or available energy, while approximate resource-allocation methods can be adopted to further reduce the computational burden.

## 5. Simulation Results

**Figure 2:**
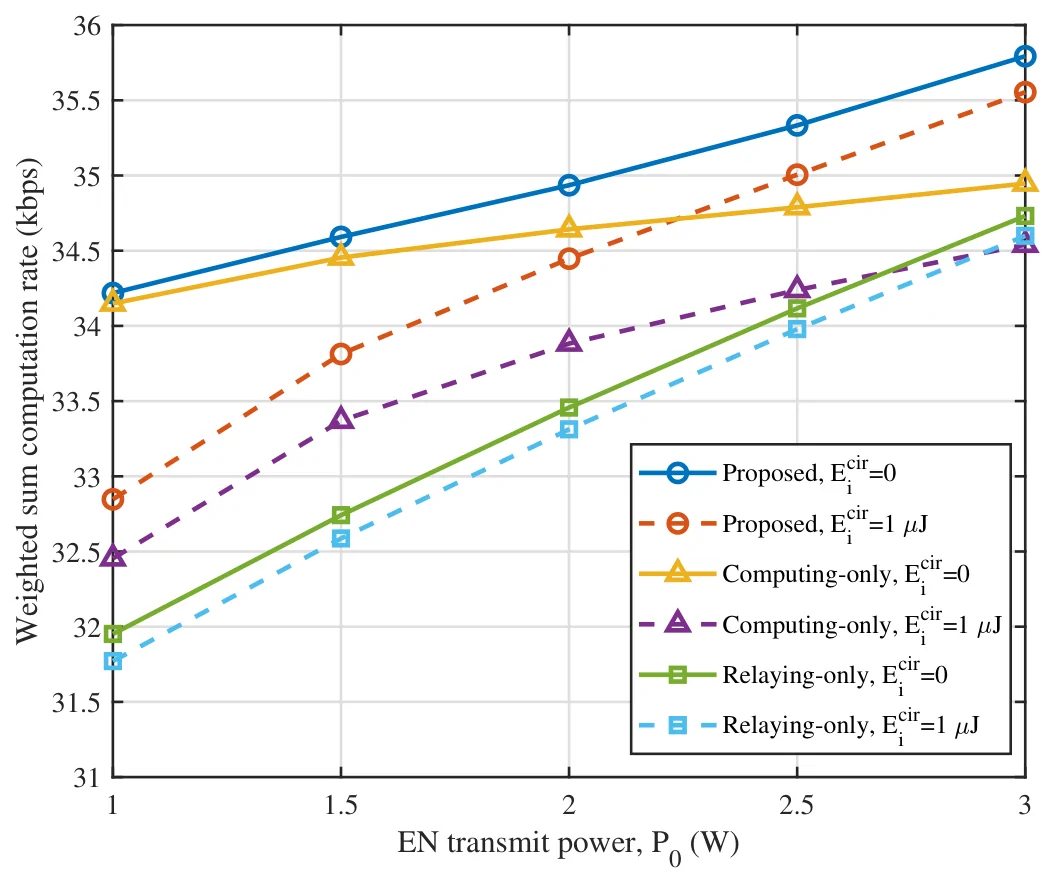
Weighted sum computation rate versus the EN transmit power under different circuit-energy settings.

In this section, the numerical results are presented to evaluate the performance of the proposed joint computing-and-relaying scheme. The paper considers a wireless-powered MEC system consisting of one EN, one ES, and N=6 users. The EN and the ES are located at (0,0) and (14,0), respectively, while the users are randomly deployed within a circular region centered at (4,0) with a radius of 2 m and a minimum inter-user distance of 0.5 m. The frame duration is normalized to T=1 s. The channel power gain of a link with distance *d* is modeled as $h=G_{A}{\left(\frac{c}{4\pi df_{c}}\right)}^{\alpha }$, where $f_{c}=915$ MHz, α=2.5, $G_{A}=2$, and $c=3\times 10^{8}$ m/s. Unless otherwise specified, the EN transmit power is $P_{0}=3$ W, the system bandwidth is B=100 kHz, the receiver noise power is $N_{0}=10^{-10}$ W, the implementation-loss factor is Γ=1, the energy-harvesting efficiency is $\eta_{i}=0.7$, and the baseline circuit-energy consumption is $E_{i}^{\mathrm{cir}}=0$ for all users, while $E_{i}^{\mathrm{cir}}=1\;\mu J$ per user per frame is separately evaluated in Figure 2.

The computation parameters are set to $\kappa_{i}=10^{-26}$, $\phi_{i}=100$ cycles/bit, and $f_{i}^{\max}=3$ MHz, while the output-to-input data ratio is $\nu_{i}=0.5$. The user weights are set to $w={\left[0.30,0.20,0.15,0.15,0.10,0.10\right]}^{T}$, representing heterogeneous computation priorities. These weights remain associated with the original user identities when different helper candidates are evaluated. Each data point is averaged over 100 independent user deployments, and all the compared schemes use the same network realizations under each parameter setting.

The proposed scheme is compared with computing-only cooperation, relaying-only cooperation, and dedicated-helper cooperation. Under computing-only cooperation, the helper processes the tasks uploaded by the task users but does not forward them to the ES; i.e., $b_{k,E}=0$ for all k∈K. Under relaying-only cooperation, the helper forwards the uploaded tasks to the ES without processing them; i.e., $b_{k,0}=0$ for all k∈K. Under dedicated-helper cooperation, inspired by [17], the helper is predetermined, while both cooperative computing and relaying are retained. Local computing remains available to every user in all the benchmarks, and the helper can process its own task locally or offload it to the ES. Except for the dedicated-helper benchmark, helper selection and all the remaining resource-allocation variables are jointly optimized.

Before presenting the subsequent parametric studies, Figure 2 evaluates the impact of practical circuit-energy consumption. The proposed, computing-only, and relaying-only schemes are compared under $E_{i}^{\mathrm{cir}}=0$ and $E_{i}^{\mathrm{cir}}=1\;\mu J$ per user per frame. Introducing nonzero circuit-energy consumption reduces the achievable WSCR of all three schemes, particularly at relatively low EN transmit power, where the fixed circuit expenditure accounts for a larger fraction of the harvested-energy budget. As $P_{0}$ increases, this relative impact gradually decreases. More importantly, the proposed joint cooperation scheme consistently achieves the highest WSCR over the entire considered range, confirming that the main comparative conclusion remains unchanged when practical circuit-energy consumption is taken into account.

**Figure 3:**
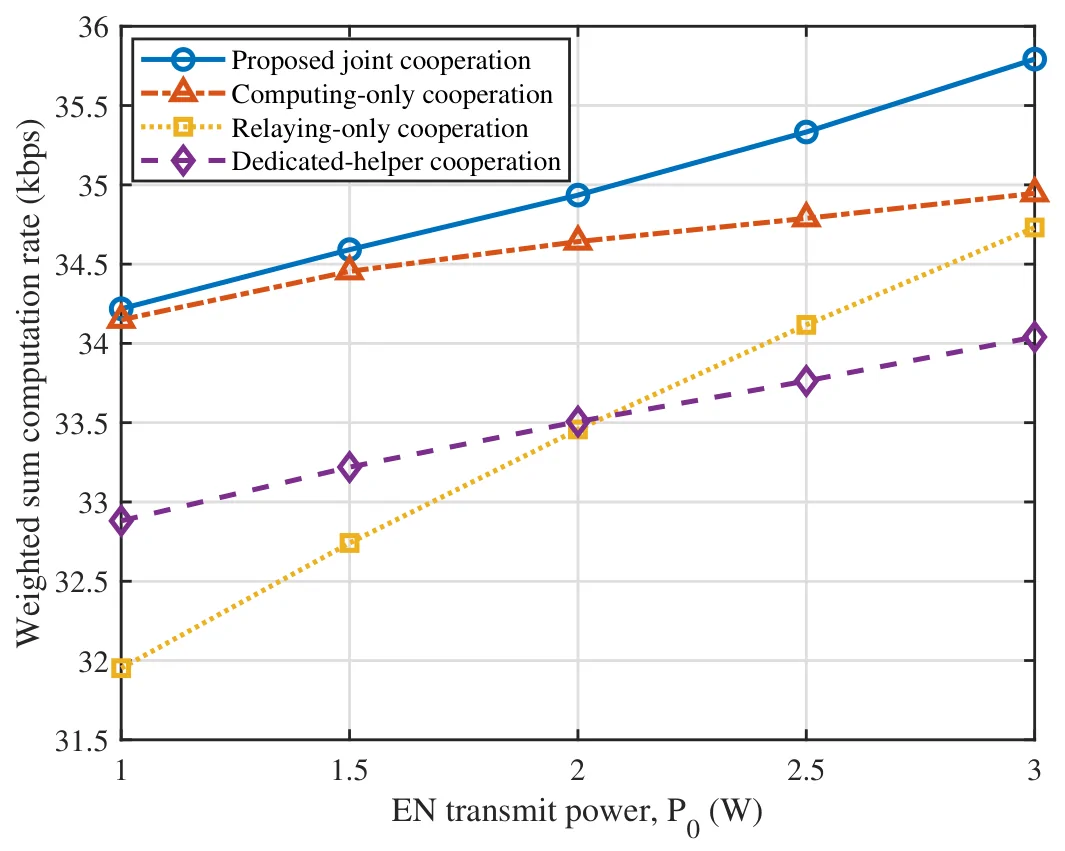
Weighted sum computation rate versus the EN transmit power $P_{0}$.

Figure 3 further examines the impact of the EN transmit power $P_{0}$ on the WSCR under the baseline circuit-energy setting. A larger $P_{0}$ increases the harvested-energy budgets of both the task users and the selected helper, thereby supporting more local computing, task uploading, helper computing, task forwarding, and result delivery. Relaying-only cooperation exhibits the strongest sensitivity to $P_{0}$ because each task bit processed at the ES consumes energy for both user-to-helper uploading and helper-to-ES forwarding. It therefore performs poorly under a tight energy budget but improves rapidly when more wireless energy becomes available. Computing-only cooperation increases more moderately because the number of task bits processed at the helper is additionally limited by its CPU capability. Dedicated-helper cooperation also improves with $P_{0}$ but remains constrained by the predetermined helper; notably, relaying-only cooperation overtakes it as the available energy increases. The proposed scheme achieves the highest WSCR throughout the considered range. As $P_{0}$ increases, it assigns more uploaded task bits to ES processing when helper computing becomes CPU-limited while retaining helper computing for task portions whose forwarding cost is relatively high. Consequently, its advantage over computing-only cooperation becomes more pronounced at higher EN transmit powers. Its consistent gain over dedicated-helper cooperation further confirms the benefit of adaptive helper selection.

**Figure 4:**
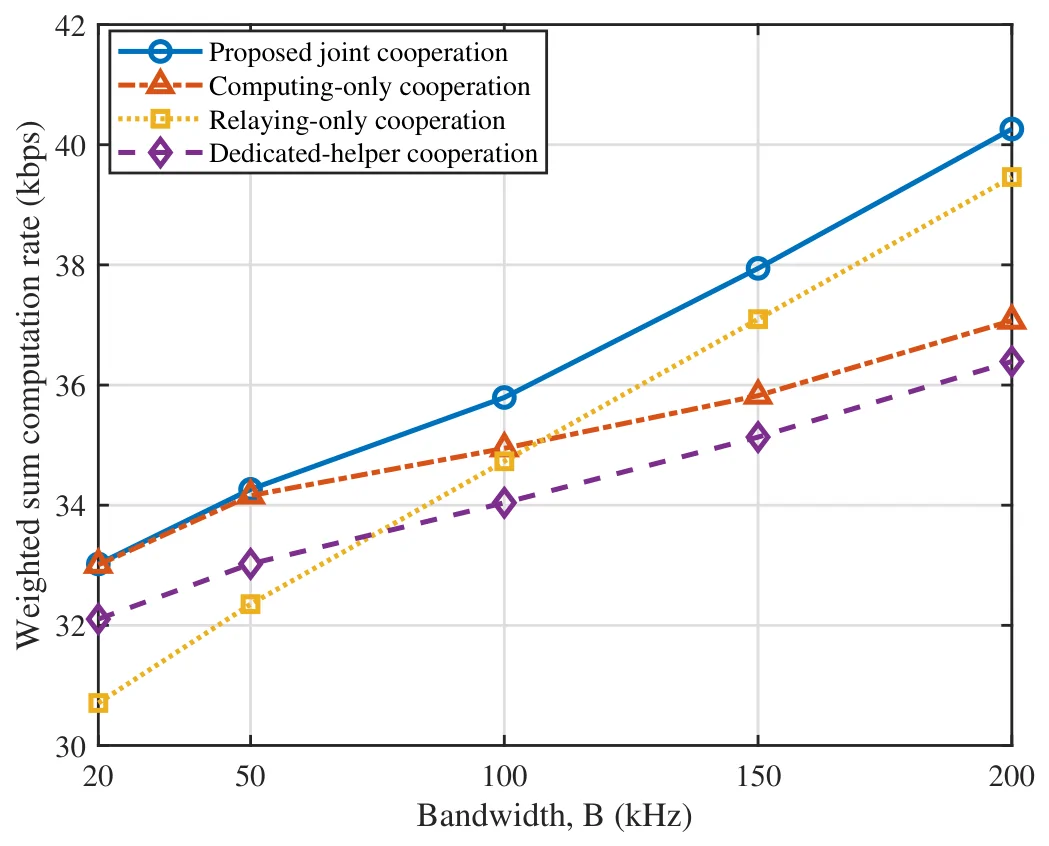
Weighted sum computation rate versus the system bandwidth *B*.

Communication bandwidth directly affects the time required for task uploading, forwarding, and result delivery. Figure 4 compares the four schemes as the system bandwidth *B* varies. Under a narrow bandwidth, computing-only cooperation nearly matches the proposed scheme, whereas relaying-only cooperation incurs a clear performance loss. This is because ES processing introduces an additional helper-to-ES transmission stage, while both cooperation modes require user-to-helper task uploading and helper-to-user result delivery. When *B* is small, these communication operations occupy a substantial fraction of the frame. The close performance of the proposed and computing-only schemes therefore indicates that helper computing is the dominant cooperative mode under a narrow bandwidth. As *B* increases, the durations required for task uploading, forwarding, and result delivery are reduced. Relaying-only cooperation consequently improves faster than computing-only cooperation and overtakes it between 100 and 150 kHz. It also overtakes dedicated-helper cooperation as the bandwidth increases, indicating that a predetermined helper limits the benefit of joint cooperation. The proposed scheme remains superior because it adaptively partitions the uploaded tasks between helper computing and ES processing instead of relying exclusively on either mode. Its additional gain over dedicated-helper cooperation further demonstrates the benefit of adaptive helper selection. The result demonstrates a transition from computing-dominant cooperation at low bandwidth to relaying-dominant cooperation at high bandwidth.

**Figure 5:**
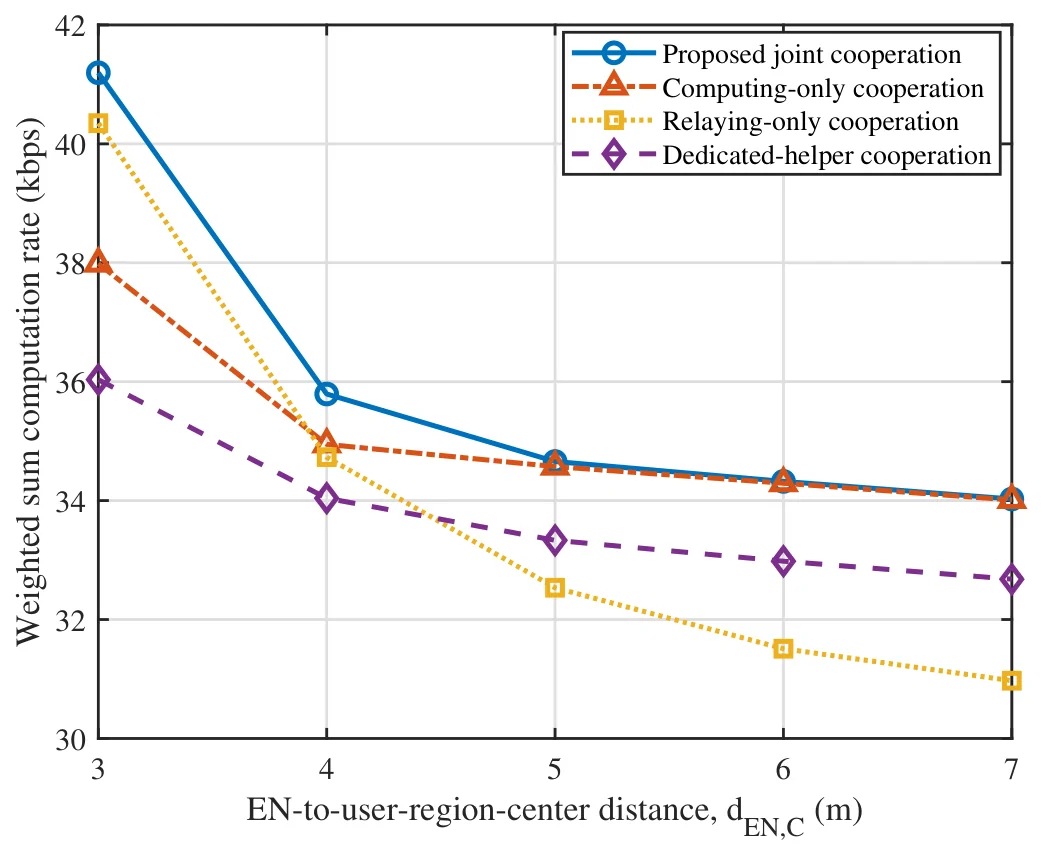
Weighted sum computation rate versus the distance $d_{\mathrm{EN},C}$ between the EN and the center of the user region.

Figure 5 examines the effect of the distance $d_{\mathrm{EN},C}$ between the EN and the center of the user region. In this experiment, the user region and the ES remain fixed. Therefore, increasing $d_{\mathrm{EN},C}$ weakens only the wireless-energy-transfer links without changing the user-to-helper or helper-to-ES information links. The resulting reduction in harvested energy lowers the feasible CPU frequencies and transmission energies of all users. Relaying-only cooperation deteriorates most rapidly because the helper must allocate part of its limited harvested energy to the additional forwarding stage in addition to processing its own task and delivering computation results. When the EN is close to the user region, this forwarding cost can be supported, and relaying-only cooperation outperforms computing-only cooperation. Their ordering reverses as $d_{\mathrm{EN},C}$ increases and the energy budget becomes tighter. Dedicated-helper cooperation degrades more slowly than relaying-only cooperation and eventually outperforms it since joint computing and relaying allow the fixed helper to reduce its reliance on energy-intensive forwarding. Meanwhile, the proposed curve gradually approaches the computing-only curve, indicating that the optimizer reduces the ES-processed task portions when the harvested energy can no longer justify the additional forwarding expenditure. The proposed scheme therefore avoids using an energy-intensive processing route under unfavorable WPT conditions while retaining an advantage over dedicated-helper cooperation through adaptive helper selection.

**Figure 6:**
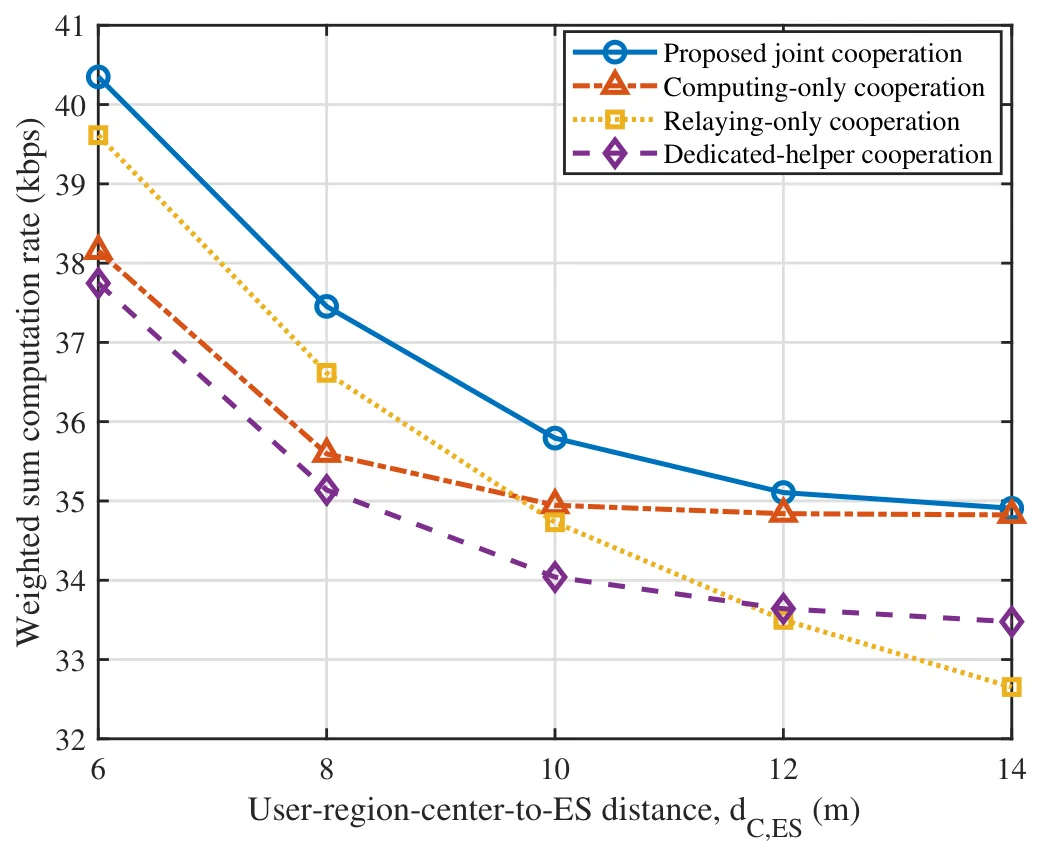
Weighted sum computation rate versus the distance $d_{C,\mathrm{ES}}$ between the center of the user region and the ES.

To isolate the helper-to-ES communication bottleneck, $d_{C,\mathrm{ES}}$ is varied while the EN and the user deployment region remain unchanged, as reported in Figure 6. Hence, the harvested-energy levels and user-to-helper channels remain statistically unchanged, whereas the helper-to-ES link weakens as $d_{C,\mathrm{ES}}$ increases. Relaying-only cooperation is particularly sensitive to this change because every task bit assigned to ES processing must pass through the degraded helper-to-ES link. The helper must therefore allocate more forwarding time or transmission energy to support the same number of task bits, reducing the resources available for other operations. Computing-only cooperation is much less sensitive because its uploaded task portions are processed at the helper and do not depend on the helper-to-ES link. Relaying-only cooperation performs better when the ES is sufficiently close but falls below computing-only cooperation between 8 and 10 m. Dedicated-helper cooperation degrades more slowly than relaying-only cooperation and overtakes it between 10 and 12 m by shifting more workload toward helper computing as the helper-to-ES link deteriorates. As the helper-to-ES link degrades, the proposed scheme increasingly benefits from helper computing, which explains why its performance approaches that of computing-only cooperation at large $d_{C,\mathrm{ES}}$. Its consistently higher WSCR than dedicated-helper cooperation also confirms the benefit of adaptive helper selection.

**Figure 7:**
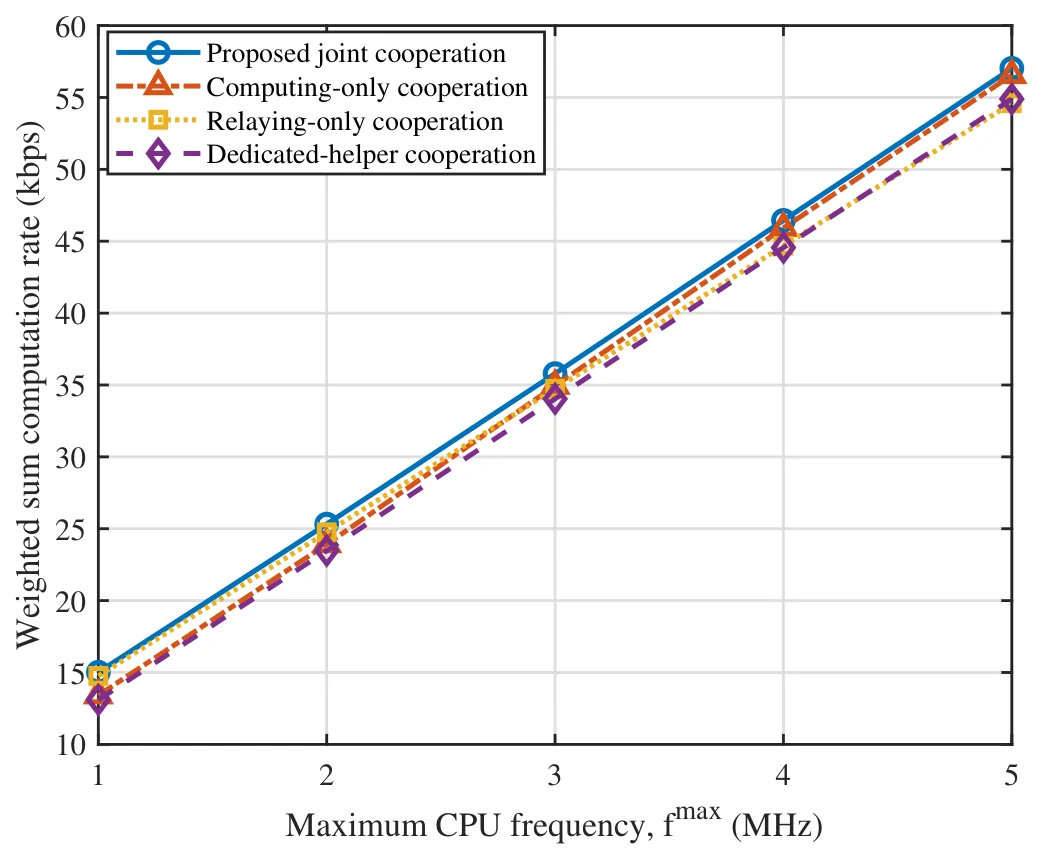
Weighted sum computation rate versus the maximum CPU frequency $f_{\max}$.

The role of computation capability is investigated next by varying the common maximum CPU frequency $f^{\max}$. The results are reported in Figure 7. Increasing $f^{\max}$ enlarges the number of CPU cycles available to all the users within one frame, thereby improving both local computing and helper computing. Over the considered range, the WSCR curves increase almost linearly because a larger CPU-frequency limit enables more local computing and helper computing. More importantly, computing-only and relaying-only cooperation exhibit a performance crossover between 1 and 2 MHz. At low CPU frequencies, the helper cannot efficiently process a large number of uploaded task bits, making ES processing more advantageous. As $f^{\max}$ increases, the helper can process more task bits during the parallel cooperation stage and avoid the time and energy required for helper-to-ES forwarding. Computing-only cooperation therefore overtakes relaying-only cooperation. Dedicated-helper cooperation also improves substantially with $f^{\max}$ but remains inferior to the proposed scheme because the helper cannot adapt to the network realization. The proposed scheme adapts its task partition accordingly: it relies more on ES processing when CPU resources are scarce and shifts more workload to the helper as the available computing capability increases. The relatively small performance gaps at high $f^{\max}$ also arise because all users receive the same CPU-frequency improvement, causing local computing to contribute an increasingly large common portion of the total WSCR.

**Figure 8:**
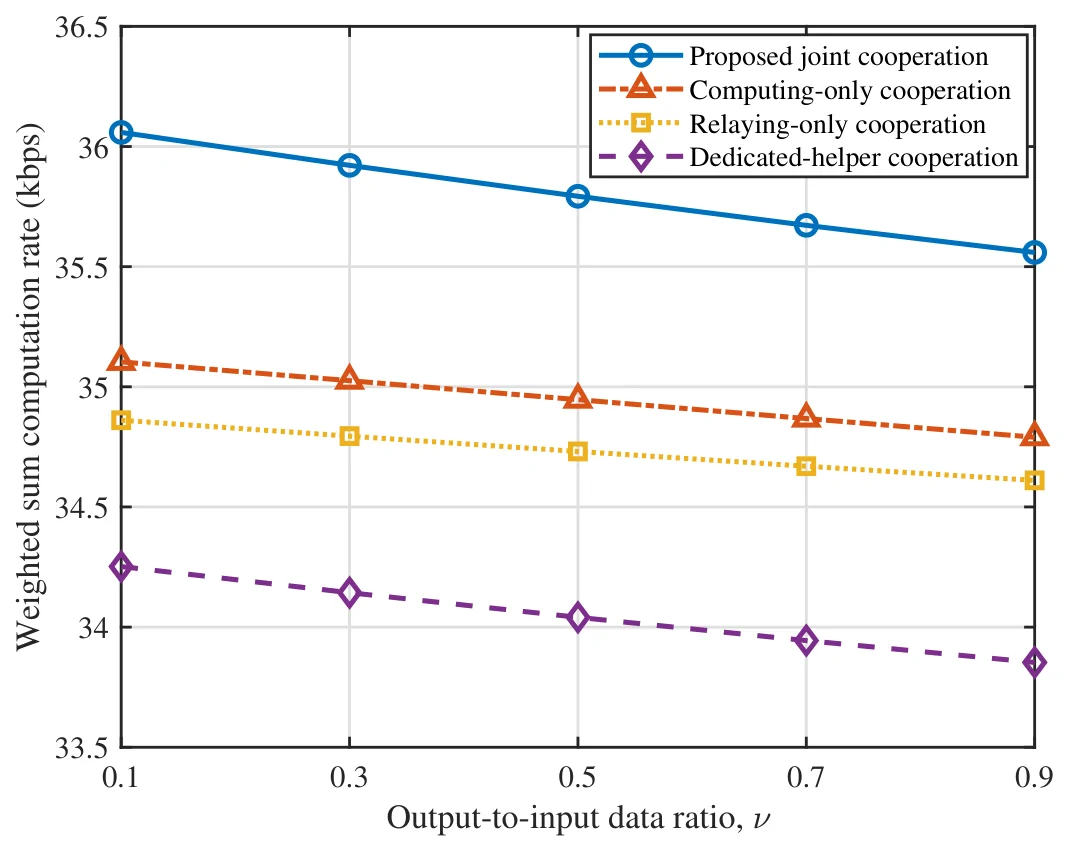
Weighted sum computation rate versus the output-to-input data ratio ν.

Figure 8 evaluates the effect of the output-to-input data ratio ν. A larger ν increases the amount of result data that must be delivered to each task user for every task bit processed at the helper or the ES. Consequently, result delivery consumes more frame time and helper transmission energy, and the WSCR decreases for all four schemes. The degradation remains moderate because locally computed task bits do not require wireless result delivery. As ν increases, each scheme can reduce the nonlocally processed task portion and allocate more workload to local computing, thereby partially compensating for the increased result-delivery overhead. The proposed scheme experiences a slightly larger absolute reduction because it processes more task bits through the helper and the ES when ν is small. A larger portion of its computation gain is therefore affected by the result-delivery constraint. Nevertheless, the proposed scheme remains superior by jointly adjusting local computing, helper computing, and ES processing according to the output-data load. Dedicated-helper cooperation exhibits a similar decreasing trend but remains inferior because the helper cannot adapt to the network realization.

**Figure 9:**
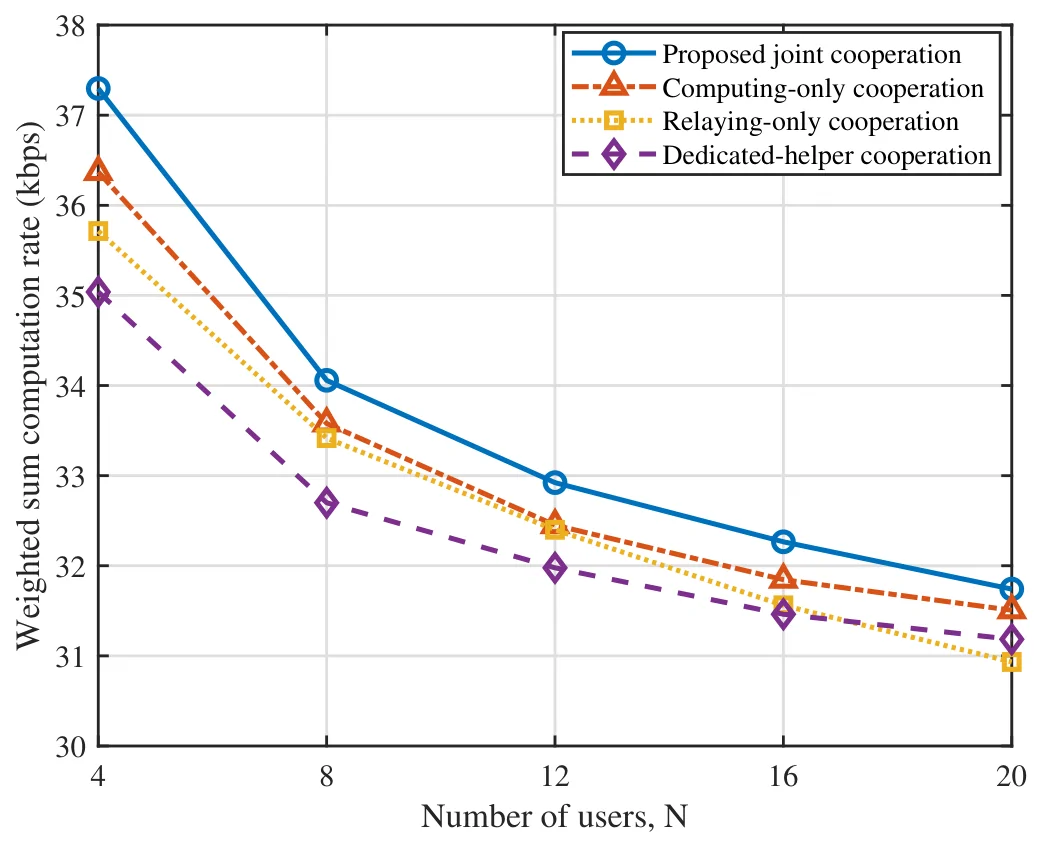
Weighted sum computation rate versus the number of users *N*.

Figure 9 evaluates the scalability of the four schemes as the number of users increases from 4 to 20. The WSCR of all the schemes decreases with *N* since more task users share the limited frame duration, harvested energy, communication resources, and helper computing capability. The proposed joint cooperation scheme consistently achieves the highest WSCR over the entire range, showing that its performance advantage is maintained as the network size increases. Among the benchmark schemes, computing-only cooperation generally performs better, while the relative performance of relaying-only and dedicated-helper cooperation varies with *N* because of their different communication and computation limitations.

**Figure 10:**
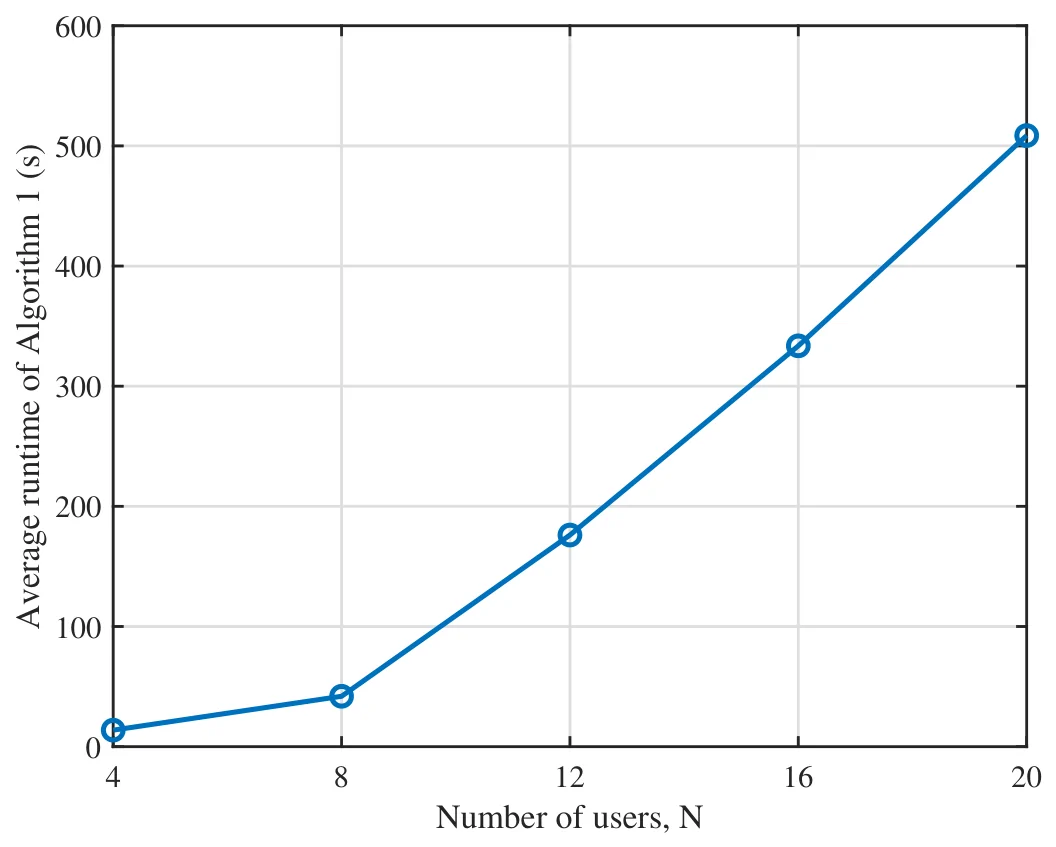
Average runtime of Algorithm 1 versus the number of users *N*.

Figure 10 evaluates the computational runtime of Algorithm 1. For each network realization, the reported runtime represents one complete execution of Algorithm 1, including all the candidate-helper subproblems and the final helper selection. Under the MATLAB R2024b with CVX 2.2 implementation used in the simulations, the average runtime increases from approximately 13 s at N=4 to approximately 510 s at N=20. The absolute runtime is implementation-dependent and is reported here to illustrate the computational growth of the exact method. This computational runtime is distinct from the transmission-frame duration T=1 s, which characterizes the communication and computation scheduling interval rather than the solver execution time. Nevertheless, if helper selection and resource allocation are recomputed online for every frame, the measured optimization latency substantially exceeds the corresponding one-second decision interval and therefore prevents direct real-time implementation of the current exact MATLAB/CVX solution. Therefore, Algorithm 1 primarily provides an exact optimal-performance benchmark, while real-time implementation requires lower-complexity helper screening or approximate resource-allocation methods.

Overall, neither computing-only cooperation nor relaying-only cooperation is uniformly preferable. Their relative performance depends on the harvested-energy budget, available bandwidth, helper-to-ES channel quality, and CPU capability. By jointly optimizing helper selection, task partitioning, and resource allocation, the proposed scheme adapts the cooperation mode to the prevailing system bottleneck and consistently achieves the highest WSCR.

## 6. Conclusions

This paper investigated joint computing and relaying for multiuser task offloading in a wireless-powered MEC system. One energy-harvesting user was selected as the helper to assist the remaining task users while processing its own task. The uploaded tasks were flexibly partitioned between cooperative computing at the helper and remote processing at the ES, with helper computing and task forwarding performed in parallel. To fully exploit this cooperation architecture, the weighted sum computation rate was maximized by jointly optimizing helper selection, task partitioning, time allocation, transmission-energy allocation, and CPU-resource allocation. For each candidate helper, the continuous resource-allocation problem was transformed into an equivalent convex problem by introducing transmission-energy variables and exploiting the perspective properties of the communication-rate and computation-energy functions. The globally optimal solution was then obtained by evaluating all the feasible helper candidates. The simulation results verify that the proposed joint computing-and-relaying scheme consistently outperforms computing-only, relaying-only, and dedicated-helper cooperation. The gain arises from its ability to adaptively balance helper computing and ES processing while selecting the helper according to the network conditions. These results demonstrate the effectiveness of jointly coordinating helper selection, task partitioning, and resource allocation in multiuser wireless-powered MEC systems.

## Data Availability

The data will be made available on request.
